# Effects of fluids on sublingual microcirculation: a point of view review

**DOI:** 10.1186/s13613-025-01607-z

**Published:** 2025-11-17

**Authors:** Arnaldo Dubin

**Affiliations:** 1 Sanatorio Otamendi, Azcuénaga 870, C1115AAB Ciudad Autónoma de Buenos Aires, Argentina; 2 Cátedras de Terapia Intensiva y Farmacología Aplicada, Facultad de Ciencias Médicas, Universidad Nacional de La Plata, La Plata, Argentina; 3 Laboratorio de Investigación Traslacional, Hospital Interzonal General de Agudos “General San Martín”, La Plata, Argentina

**Keywords:** Microcirculation, Fluids, Colloids, Crystalloids, Videomicroscopy

## Abstract

**Background:**

Fluids are a key component of shock resuscitation. Nevertheless, their microvascular effects are complex. In fluid responsive patients, fluids may increase tissue perfusion because of the increase in cardiac output. Since shock states are characterized by a partial loss of the coherence between systemic hemodynamics and microcirculation, the increase in cardiac output does not guarantee improvements in tissue perfusion. Furthermore, the administration of fluids carries risks of hemodilution and tissue edema that can dampen microcirculation. Regarding this, colloid solutions have some theoretical advantages and experimental support. Despite its relevance, few clinical studies have evaluated the effects of fluids on sublingual microcirculation—the more clinically accessible territory for videomicroscopy. This review analyzes physiological bases and experimental and clinical evidence about the complex microvascular effects of fluids.

**Main text:**

We found eight observational and four controlled trials carried out on critically ill and surgical patients addressing the effects of fluids on sublingual microcirculation. Most showed that fluid resuscitation can improve microcirculation, especially in the presence of fluid responsiveness and tissue hypoperfusion. Concerning the controlled trials that compared different solutions, one study failed to show benefits of hypertonic over isotonic hydroxyethyl starch, while another found improved microcirculation after early goal-directed therapy with hydroxyethyl starch than with 0.9% NaCl. Since both studies included a small sample, the results are inconclusive. The third trial, which recruited 100 septic patients, concluded that albumin was superior to balanced solution; however, this conclusion is flawed by methodological problems. Some experimental studies also showed controversial results. Some studies which suggested benefits of albumin, hydroxyethyl starch and high viscosity solutions could not be properly replicated in patients. Superiority of balanced crystalloids over 0.9% NaCl was not consistently demonstrated in basic research. Moreover, some clinical and experimental studies have severe limitations, such as the use of inadequate analysis of microcirculation and compression artifacts in the video acquisition.

**Conclusions:**

Fluid administration probably improves sublingual microcirculation when tissue perfusion is altered and cardiac output increases. The superiority of any solution for this purpose has not been clearly demonstrated. High-quality studies are needed to clarify the effects of different solutions on sublingual microcirculation.

## Introduction

Fluids are the cornerstone of resuscitation in shock. The goal of fluid resuscitation is to improve cardiac output and tissue perfusion. The relationship of cardiac output to tissue perfusion, however, is not straightforward [1]. There is physiological coherence between systemic hemodynamic and microcirculation. Severe abnormalities in cardiac output and blood pressure usually result in microvascular compromise. In such conditions, normalization of systemic hemodynamics generally improves microcirculation. After resuscitation, however, coherence might be lost and dissociation between systemic cardiovascular variables and microcirculation can occur, especially in septic shock.

The outcome of patients with septic shock is not related to the magnitude of cardiac output but to tissue perfusion. Thus, sublingual microvascular alterations are more severe in nonsurvivors than in survivors of septic shock [2, 3]. Nonsurvivors exhibit lower values of perfused capillary density, proportion of perfused microvessels, and red blood cell velocity, along with increased flow heterogeneity than survivors. [4] These derangements are similar in hyperdynamic or normodynamic septic shock, and are not related to the actual values of cardiac output or blood pressure [5]. Since the final goal of resuscitation is the normalization of tissue perfusion and not of cardiac output, the monitoring of the effects of fluids on microcirculation is a key issue.

Given that the visualization of sublingual mucosa by videomicroscopy is the most commonly used approach for the direct assessment of microcirculation, the purpose of this review is to summarize the scientific evidence related to the effects of fluids on sublingual microcirculation. We will discuss the physiological regulation of microcirculatory perfusion, the ability of volume resuscitation to improve microvascular flow, the determinants of this response, the equivalence of different solutions for this goal, and the relevance of the sublingual window to reflect the effects of solutions on other microvascular beds. Another relevant issue, the effect of fluids on glycocalyx, has been comprehensively addressed elsewhere and will not be included in this review [6–9]. This review does not include a discussion about the effects of blood products on microvascular perfusion.

## Theoretical considerations about the beneficial and detrimental effects of fluids on microcirculation

Fluid administration might be useful to increase microvascular perfusion in patients with fluid responsiveness. In this case, solutions increase mean systemic pressure and its gradient to central venous pressure, which acts as the driving pressure to venous return [10]. Thus, the increased cardiac preload results in improved cardiac output. The subsequent impact of the augmented systemic flow on microcirculation is controversial and depends on the coherence between macro- and microcirculation [1]. Besides, each microvascular bed could exhibit a particular and variable response.

On the other hand, the increase in hydrostatic capillary pressure induced by fluid administration might be associated with tissue edema. Transcapillary fluid exchange has classically been described by Starling’s principle, which attributes the balance between filtration and reabsorption to opposing hydrostatic and oncotic forces across the capillary wall. In this traditional view, fluids are filtered at the arteriolar end and reabsorbed at the venular end. The revised Starling principle, however, identifies the endothelial glycocalyx as the primary determinant of vascular permeability and oncotic pressure gradients [11]. In this model, most filtered fluid returns to the circulation via the lymphatic system, rather than through venular reabsorption. The effective oncotic gradient lies between the plasma and the subglycocalyx space, which contains minimal protein. Given that the oncotic difference is already large under normal conditions, increasing plasma albumin concentration only slightly increases this gradient. This explains the limited capacity of colloids to sustain intravascular volume. When the glycocalyx is degraded, as occurs in sepsis or inflammation, capillary permeability rises and the reflection coefficient declines, allowing colloids to extravasate almost as freely as crystalloids, thereby negating any theoretical advantage of albumin.

Tissue edema might decrease vascular density and increase oxygen diffusional distance from capillaries to mitochondria. As proof of concept, a study performed in patients during the postoperative de-escalation period of cardiac surgery, suggested that the recovery of functional capillary density is partly associated with the removal of excess fluid and tissue edema produced by diuretic therapy [12]. Although crystalloid solutions might induce higher fluid extravascular losses than colloid solutions because of crystalloid’s higher volume of distribution, a meta-analysis showed that mean crystalloid/colloid ratio across all the studies included was only 1.5 [13]. In some controlled studies, there were no differences in the volume of crystalloids or colloids required to meet hemodynamic goals [14, 15]. This finding is explained by endothelial failure, which produces increase in permeability to high molecular weight substances, as well by the revisited Starling principle [11, 16].

Moreover, the increase in venous pressure could potentially contribute to reduce tissue perfusion. Microcirculatory perfusion pressure is traditionally defined as the difference between precapillary and venular pressure; therefore, excessive increments in venous pressure could decrease microvascular flow [17]. In contrast, if we consider that tissue perfusion pressure is the gradient between mean arterial pressure and the critical closing pressure, tissue perfusion should be independent of venous pressure in most circumstances [18]. Critical closing pressure is the intraluminal pressure at which vessels collapse; it is considered as the effective back-pressure to arterial flow, independent of downstream capillary and venous pressures. Critical closing pressure is usually higher than mean systemic filling pressure. The difference between critical closing pressure and mean circulatory filling pressure is so-called the “vascular waterfall”, as flow over the edge is independent of how far it falls subsequently. According to this hypothesis, tissue perfusion is only affected by venous pressure in absence of waterfall effect [19]. Similar critical closing and mean systemic pressures can result from extreme arterial vasodilation or very high venous pressure.

Another potential detrimental effect of fluids on microcirculation is the development of compartmental syndrome in capsulated organs, such as the kidney [20, 21].

Finally, fluids can induce hemodilution. The effects of hemodilution on cardiac output and on microcirculation are complex and mainly determined by changes in viscosity. In physiological conditions, the main determinant of blood viscosity is hematocrit; as it is reduced, the weight of plasma viscosity increases [22]. Reduced viscosity decreases systemic vascular resistance and increases cardiac output. Additional mechanisms of increased systemic flow are increases in venous return and sympathomimetic activity leading to tachycardia and positive inotropic effect [23]. Even though, the relationship between viscosity and vascular resistance is not linear. Small, acute decrease in hematocrit (∼10%) elevates blood pressure and vascular resistance [24]. This phenomenon results from reduced blood viscosity, which decreases shear stress on the vascular wall and thus decreases the production of nitric oxide. Further falls in hematocrit are followed by reductions in systemic vascular resistance.

**Fig. 1:**
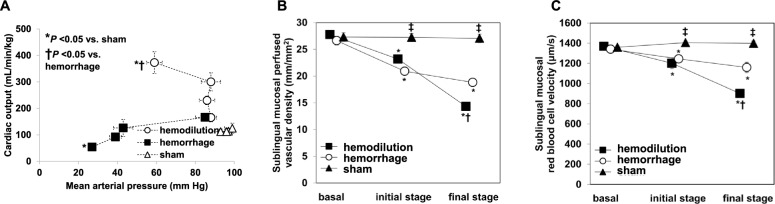
Dissociation between systemic hemodynamics and microcirculation during progressive hemodilution. Comparison of the behavior of systemic cardiovascular and microcirculatory variables during stepwise hemodilution and hemorrhage. In hemodilution, there were progressive increases in cardiac output along with reductions in microvascular perfusion. Panel A: Cardiac output and mean arterial blood pressure. Panel B: Sublingual perfused vascular density. Panel C: Sublingual red blood cell velocity

**Fig. 2:**
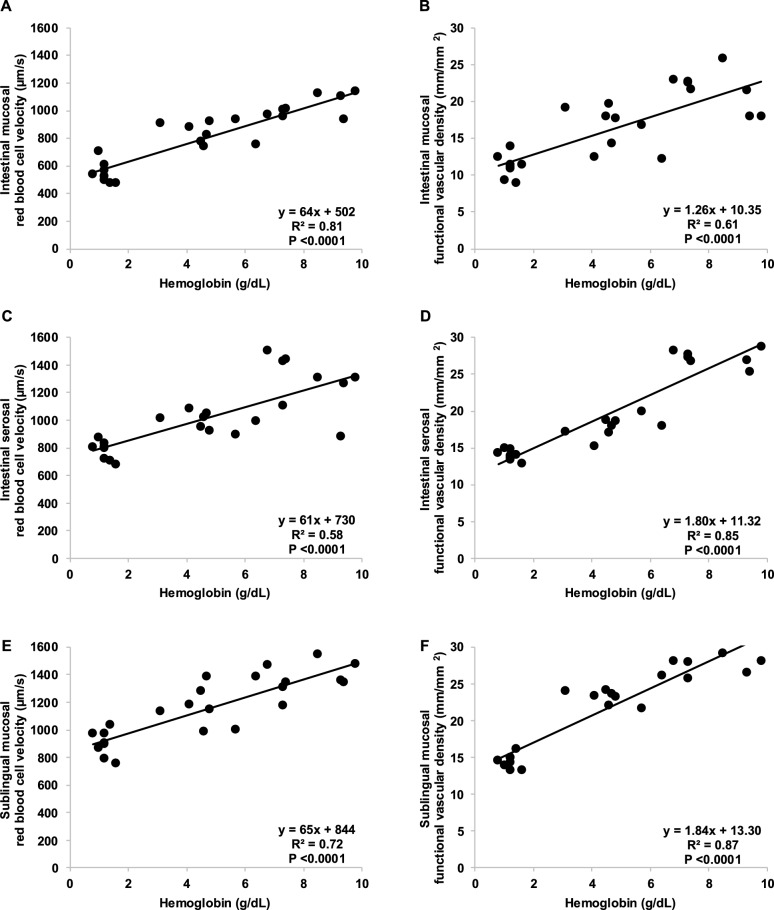
Hemodilution is associated with progressive decline in microcirculatory perfusion. Hb levels is linearly correlated with intestinal mucosal red blood cell velocity (**A**), intestinal mucosal functional capillary density (**B**), intestinal serosal red blood cell velocity (**C**), intestinal mucosal functional capillary density (**D**), sublingual mucosal red blood cell velocity (**E**), and sublingual mucosal functional capillary density (**F**)

The effects of hemodilution on microcirculation are even more complex. A consensus conference considered the hyperdynamic capillary flow as the typical microvascular abnormality of hemodilution [25]. This concept, however, is not clearly supported by clinical or experimental evidence. Hemodilution up to 15% of hematocrit was linked to improved microcirculation [26]. Higher level of hemodilution, however, induces progressive and severe alterations in microvascular flow that are paradoxically associated with high cardiac output. Extreme hemodilution is the most paradigmatic condition of dissociation between systemic hemodynamics and microcirculation (Fig. 1). In sheep exposed to stepwise hemodilution, there were reductions in total and perfused vascular densities, in the proportion of perfused vessels, and in the red blood cell velocity. These changes occurred in intestinal mucosa and serosa, and in sublingual mucosa [27]. All microcirculatory variables were strongly correlated with hemoglobin level (Fig. 2). Yet, the behavior of microcirculation might differ among some territories. Skin red blood cell velocity exhibits a biphasic response to hemodilution, with an early increase followed by a subsequent decrease, while functional capillary density initially remains unchanged and then progressively declines [28]. Differently to the behavior in most microvascular beds, red blood cell velocity gradually increases in heart and central nervous system during hemodilution [29, 30].

The detrimental microvascular effects of hemodilution are explained by the reduction in blood viscosity. This phenomenon reduces vascular wall shear stress and production of endothelia-dependent vasodilators such as nitric oxide, and causes vasoconstriction and decreased perfused vascular density [31]. Viscosity is a critical factor to maintain microcirculatory patency. Tissue oxygenation might be additionally threatened by reductions in microvascular hematocrit. Fåhræus effect determines that microvascular hematocrit decreases with decreasing capillary diameter. Partially related to Fåhræus effect, Fåhræus-Lindqvist effect establishes that viscosity decreases with decreasing capillary diameter [32]. Indeed, extreme hemodilution induces severe derangements in both tissue perfusion and oxygenation. It has been suggested that tissue oxygenation is more compromised due to the redistribution of flow rather than by the deficit in the oxygen carrier [33]. Therefore, the administration of solutions of higher viscosity might improve tissue perfusion and oxygenation during severe hemodilution, in which plasma viscosity is a relevant determinant of total blood viscosity.

A study performed in patients with trauma failed to find consistent relationship between plasma viscosity and microcirculatory variables [34]. Since patients received a median volume of 2000 (IQR 0–3000) mL over 48 h, probably none had extreme hemodilution. As plasma viscosity acts as a critical determinant of blood viscosity in severe hemodilution only, the results were expectable.

**Fig. 3:**
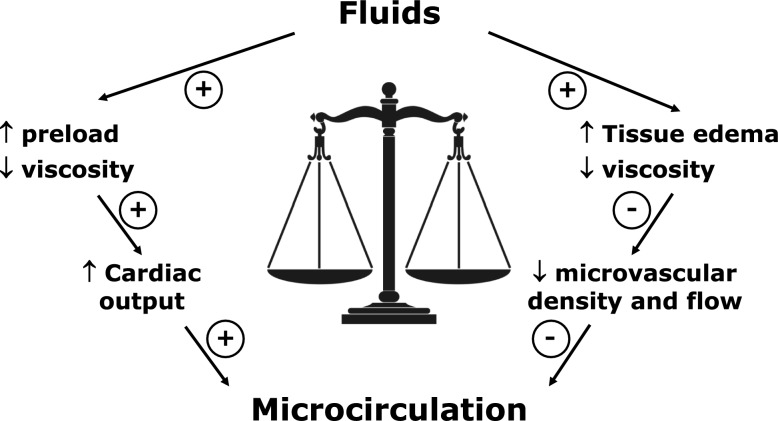
Schematic effects of fluids on microcirculation

In brief, fluid administration might be a double-edge sword for microcirculation (Fig. 3). The final effects result from the interplay of the multiple determinants of microcirculatory flow.

## Can fluids improve sublingual microcirculation?

Many clinical and experimental studies showed that different solutions can increase microvascular perfusion. However, it depends on the underlying pathophysiological conditions, such as the presence of fluid responsiveness, and/or the presence of tissue hypoperfusion. There are also controversies about the superiority of any particular type of fluid. A systematic review analyzed the results of six randomized controlled trials and nine prospective observational studies including 813 septic patients, in whom fluid resuscitation or blood transfusion were aimed at improving sublingual microcirculation [35]. Given the great heterogeneity between studies, a meta-analysis was not carried out, and the authors concluded that the results failed to show a consistent, measurable improvement in sublingual microcirculation following fluid resuscitation. In spite of this conclusion, the analysis of controlled and observational studies—some of them not considered, or even misinterpreted in the aforementioned review—showed the ability of volume expansion to improve sublingual microcirculation.

**Fig. 4:**
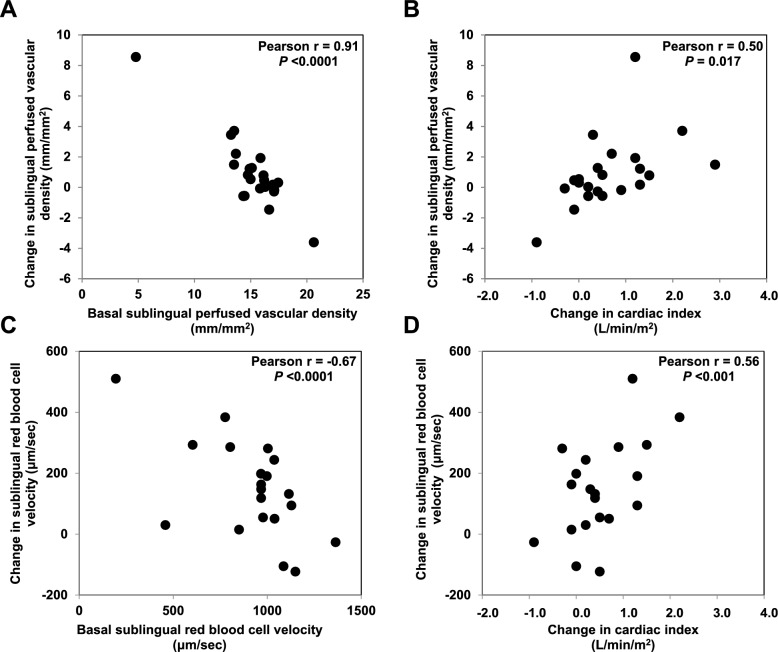
The effects of a fluid challenge on sublingual microcirculation in septic patients are dependent on the increase in cardiac output and the basal state of microcirculation. Correlations between changes in sublingual perfused vascular density and basal perfused vascular density (**A**), changes in sublingual perfused vascular density and changes in cardiac output (**B**), changes in sublingual red blood cell velocity and basal red blood cell velocity (**C**), changes in sublingual red blood cell velocity and changes in cardiac output (**D**)

We analyzed eight observational and four controlled studies focused on the microvascular effects of fluids (Table 1). The beneficial effects of fluids seem more evident in the presence of fluid responsiveness and/or tissue hypoperfusion. A study cited in the mentioned systematic review as an example of the failure of fluids to affect microcirculation included twenty-four patients with septic shock randomized to receive 250 mL of 7.2% NaCl/6% hydroxyethyl starch (HES) or 500 mL of 6% HES [36]. Even though sublingual microcirculation remained unchanged in both groups, improvements in microvascular flow index were noticed in patients of both groups who showed fluid responsiveness. In patients with sepsis and septic shock and high values of respiratory pulse pressure variation (22 ± 5%), either passive leg raising or volume expansion with 500 mL of 0.9% NaCl or HES 130/0.4 increased sublingual microcirculatory perfusion [37]. Accordingly, a bolus of 250 mL of HES 130/0.4 produced favorable effects on most sublingual microcirculatory variables in septic patients who simultaneously responded with increased stroke volume, whereas such effects were not found in nonresponders [38]. In surgical patients, alterations in sublingual microcirculation were linked to preload dependence and were corrected after a fluid challenge [39]. In another controlled trial that compared two strategies of fluid resuscitation in patients with high-risk abdominal surgery, a more aggressive management was associated with higher cardiac output and improved sublingual microcirculation [40]. Another observational study performed in critically ill patients found that infusion of a sodium acetate-based isotonic solution rises sublingual microvascular flow index and the proportion of perfused vessels only in stroke volume responders with clinical signs of tissue hypoperfusion [41]. Moreover, the effects of 10 mL/kg of 6% HES 130/0.4 on sublingual red blood cell velocity and perfused vascular density were strongly correlated with the basal state of microcirculation and the magnitude of fluid responsiveness [42]. Thus, the beneficial actions of fluid boluses were more pronounced in patients with deeper hypoperfusion and higher increase in cardiac output (Fig. 4). In the early phase of sepsis, the administration of 1,000 mL of Ringer lactate (n=29) or 400 mL of 4% albumin (n=31) increased the sublingual proportion of perfused vessels and the perfused vascular density, but this effect was lost in later stages [43]. Notably, no relationship between these effects and those in cardiac index or mean arterial pressure were observed. In early sepsis (< 24 h.), the microvascular effects were dependent on the basal state of microcirculation. Another study showed that the infusion of Ringer acetate/malate was followed by improvements in sublingual microcirculation in low-grade hemodilution [26]. Higher degrees of hemodilution worsened microvascular perfusion.

**Table 1:** Clinical studies evaluating the effects of fluids on sublingual microcirculation

Author	Design	Fluid	Patients	Analysis	Main results	Observations
Hahn et al. [25]	Single-center prospective observational study	Ringer acetate/malate to generate hemodilution of 10, 20, or 30%	Surgery (n=20)	AVA 4.3 software	Microcirculation improved with hemodilution<15% and worsened with>15%	Use of nonvalidated software Lack of CO measurements
Van Haren et al. [35]	Single-center randomized controlled study	250 mL 7.2% NaCl/6% HES vs. 500 mL 6% HES	Septic shock (n=24)	AVA 3.0 software	No differences between groups in any microvascular variable MFI increased only in fluid responders	No data about the response of the rest of the microvascular variables in responders and nonresponders
Pottecher et al. [36]	Two-center prospective observational study	500 mL normal saline or 500 mL 6% HES 130/0.4	Sepsis (n=25)	AVA 1.0 software	Microcirculation improved in fluid responders	Gross compression artifacts in videos posted as supplementary material
Klijn et al. [37]	Single-center prospective observational study	250 mL 6% HES 130/0.4	Sepsis (n=35)	AVA 3.0 software	Most microvascular variables improved but only in fluid responders	Gross compression artifacts in videos posted as supplementary material
Bouattour et al. [38]	Single-center prospective observational study	500 mL 0.9% NaCl	Abdominal surgery (n=17)	AVA 1.0 software	Microcirculation improved in fluid responders	
Coeckelenbergh et al. [39]	Two-center randomized controlled study	250 mL balanced crystalloid and 5% albumin	Abdominal surgery (n=86)	AVA 4.3 software	Microcirculation improved in patients more aggressively resuscitated	Use of nonvalidated software
Pranskunas et al. [40]	Single-center prospective observational study	500 mL 0.9% NaCl or 6% balanced HES 130/0.4	Critically ill patients with impaired perfusion (n=50)	AVA 3.0 software	Microcirculation improved only in patients with MFI<2.6 and fluid responsiveness	
Edul et al. [41]	Single-center prospective observational study	10 mL/kg of 6% HES 130/0.4	Abdominal sepsis (n=22)	AVA 3.2 software	Improvement in microcirculation related to basal state and fluid responsiveness	
Ospina-Tascon et al. [42]	Single-center prospective observational study	1,000 mL Ringer lactate or 400 mL 4% albumin	Sepsis (n=60)	Analysis by eye	Microcirculation improved in early (<24 h) but not in late sepsis (>48 h) In early sepsis, improvement related to microvascular basal state but not to CO	
Stens et al. [43]	Single-center randomized controlled study	Ringer lactate and colloids	Abdominal surgery (n=31)	AVA 3.0 software	Similar microcirculation in both groups despite more volume of fluids and higher CO in the intervention group	Microvascular variables at baseline were only slightly altered
Cusack et al. [44]	Single-center randomized controlled study	20% albumin vs. isotonic sodium lactate	Sepsis (n=100)	AVA 4.3 software	20% albumin marginally increased some surrogates of vascular density while others remained unchanged No changes in crystalloid group	Use of nonvalidated software Nonsignificant time-by-group interactions

*SDF* sidestream dark field videomicroscope, *HES* hydroxyethyl starch, *MFI* microvascular flow index, *CO* cardiac output

Differently to the results of previous studies, a small randomized controlled trial failed to find beneficial effects of fluids on microcirculation [44]. Patients with abdominal surgery were hemodynamically managed according to the changes in either respiratory pulse pressure variation and cardiac output or mean arterial pressure. Despite the higher volume of infused fluids and increased cardiac output in intervention than in control group, microcirculatory variables did not differ between groups. Although the results might be explained by failure of fluid resuscitation, microcirculatory variables at baseline were only slightly altered in both groups. A controlled trial performed in septic patients with fluid responsiveness compared the infusion of 20% albumin with isotonic sodium lactate solution [45]. In the albumin group, some surrogates of sublingual vascular density marginally increased while others remained unchanged, which is difficult to explain. In contrast, the proportion of perfused vessel was not modified. In the control arm of the study, no microcirculatory variable was affected.

In summary, despite the possible loss of coherence between systemic cardiovascular and microvascular variables in shock patients, clinical evidence suggests that fluid administration can improve sublingual microcirculation, especially in presence of severe tissue hypoperfusion and high preload-dependence. Table 1 summarizes the main findings of clinical studies addressing the effects of fluids on sublingual microcirculation. A randomized controlled trial that assessed the effectiveness and feasibility of fluid resuscitation directed by microcirculation monitoring in patients with septic shock is not discussed because it does not provide basic information about the assessment of microcirculation [46].

Besides clinical studies, many experimental models also showed that fluids can improve microcirculation in sublingual mucosa as well as in other microvascular beds. These effects have been observed in models of hemorrhagic and septic shock. The increase in tissue perfusion may differ according to the experimental situation, the microcirculatory territory, and the fluid utilized. As in clinical studies, the coherence between macro- and microcirculation can be altered. In a large animal model of complex traumatic injury and hemorrhagic shock, resuscitation with blood products or 0.9% NaCl targeted at systolic blood pressure resulted in wide interindividual variations in both macro- and microcirculatory flow variables [47]. Although microcirculatory flow initially followed cardiac output, the coherence was lost during late resuscitation to some extent.

## Effects of particular fluid solutions

Beyond the different ability to expand intravascular volume, fluids have particular effects. Each of the molecules of a solution has potential to induce either beneficial or adverse effects that can directly or indirectly affect microcirculation. Moreover, the demonstration of beneficial microvascular actions in specific clinical or experimental settings might be overcome by evidence showing detrimental effects on clinical outcomes. Unfortunately, few randomized clinical trials have compared the ability of different solutions to improve microcirculation.

### Hydroxyethyl starches

Experimental studies showed that HES 130/0.4 is better than saline solution for this purpose. In rabbits with hemorrhagic shock, HES 130/0.4 was superior to normal saline for recruitment of sublingual microcirculation, but this effect might have been secondary to better resuscitation of systemic hemodynamics rather than a specific effect on microcirculation [48]. However, in a septic model, HES, compared to 0.9% NaCl, decreased microvascular leukocyte adherence and increased functional capillary density, despite both fluids showing similar systemic cardiovascular effects [49]. Thus, an antinflammatory effect of HES might have been involved [50]. Based on these results, a small randomized controlled trial was carried out comparing HES 130/0.4 and 0.9% NaCl during the early goal-directed therapy of septic patients [51]. The results showed that HES 130/0.4 significantly improved sublingual microcirculation. Nevertheless, randomized controlled trials showed that HES is not superior to crystalloids for hemodynamic resuscitation of shock, has toxic effects, and increases mortality of septic patients [52]. Consequently, this solution should not be used in critically ill patients.

### Albumin

Albumin is another attractive molecule for the resuscitation of microcirculation. Endogenous albumin plays a major role in the maintenance of microvascular function. The binding of albumin to the glycocalyx reduces hydraulic conductivity across the vascular barrier, avoids its degradation, and enhances the transmission of shear stress [53]. Albumin is the major determinant of plasma colloid osmotic pressure. In addition, albumin transports sphingosine-1-phosphate which has protective effects on the endothelium, acts as a free radical scavenger, and has immunomodulatory and anti-inflammatory effects. In experimental hemorrhagic shock, the infusion of 5% albumin was associated with preservation of glycocalyx and permeability compared with Ringer lactate or 0.9% NaCl; however, the differences in red blood cell velocity and venular diameter between both fluids were subtle [54]. In a model of rat endotoxemia, infusions of 4% or 20% albumin reached higher values of skeletal muscle perfused vascular density and red blood cell velocity than 0.9% NaCl [55]. Different to other colloids, human serum albumin is safe for volume expansion in critically ill patients. Nevertheless, controlled studies failed to show beneficial effects of 5% human albumin solution on different outcomes when compared to crystalloids [56, 57]. A single-center randomized controlled trial assigned 100 patients with positive fluid responsiveness to receive boluses of either 20% albumin bolus or sodium lactate and assessed the effects on sublingual microcirculation [45]. Although the authors concluded that 20% albumin improves microcirculation this is not clearly supported by the results, since microvascular changes were minor and significant time-by-group interactions were not shown for any microcirculatory variable. Therefore, the clinical usefulness of albumin for the recruitment of microcirculation in septic patients remains uncertain.

Albumin-derived molecules with higher molecular weight might have an increased ability to improve microcirculation. Human serum albumin conjugated with polyethylene glycol (PEG) is a plasma expander with increased viscogenic and microcirculatory effects. Some of its actions could be linked to higher availability of nitric oxide [58]. In an experimental study of hemodilution followed by hemorrhagic shock, it resulted in improved survival and better microcirculation compared to HES 130/0.4 [59]. In an endotoxin model of sepsis, PEG-conjugated albumin also exhibited better microvascular effects than dextran [60]. Polymerized albumin was superior to albumin to restore macro and microcirculation, and to prolong survival in experimental models of endotoxemia in mice and of fecal peritonitis in hamsters [61]. Recently, a “protein cocktail”—a mixture of approximately 40% human serum albumin, 35% transferrin, 10% haptoglobin, and 5% hemopexin—showed microcirculatory effects comparable to those of whole blood in experimental hemorrhagic shock [62]. Nevertheless, neither of these albumin derived molecules have been clinically evaluated.

### High-viscosity solutions

Unlike experimental studies, in which the use of plasma expanders with increased viscosity has marked advantages for microcirculation, their administration to critically ill patients could be a different pathophysiological situation. In animal models of extreme hemodilution, the limiting factor to tissue oxygenation might not be the decrease in the oxygen-carrying capacity but the decline of microvascular function due to decreasing functional capillary density. Thus, high-viscosity solutions might probably be useful because blood viscosity mainly depends on plasma viscosity. In patients without critical degrees of hemodilution, blood viscosity is mainly determined by hematocrit. Consequently, increases in plasma viscosity might not affect blood viscosity and fail to increase microvascular perfusion.

### Hypertonic solutions

Another point of controversy is the usefulness of hypertonic solutions. Some experimental studies suggested that hypertonic solutions induce favorable actions on microcirculation, compared to isotonic solutions [63–65]. Yet, the experimental evidence is contradictory [66]. In the resuscitation of hemorrhagic shock, gelatin, 6% HES 130/0.4 and NaCl 7.2%-6% HES 200/0.5) similarly normalized sublingual microcirculation [67]. There are also opposite reports about the microvascular effects of hypertonic sodium lactate solution [63, 68]. And, most importantly, hypertonic saline solutions do not affect survival in patients with hypovolemic shock [69]. Only one controlled study assessed the benefits of hypertonic solutions on sublingual microcirculation: twenty-four patients with septic shock were randomized to receive 250 mL 7.2% NaCl/6% HES or 500 mL 6% HES [36]. Even though hypertonic solution improved cardiac contractility and vascular tone compared with isotonic fluid and reduced the need for ongoing fluid resuscitation, there were no differences in the effects on sublingual microcirculation. Thus, the evidence does not support the use of hypertonic solutions for microcirculation improvement.

### Other solutions

Multiple studies compared the effects of different solutions on microcirculation in experimental models of hemorrhagic and septic shock. A systematic review of preclinical studies assessed the optimal fluid for the recruitment of microcirculation after hemorrhagic shock [70]. Some studies reported the importance of the presence of hemoglobin, as well as molecules of high osmotic power and viscosity for optimal restoration of microcirculation. Others emphasized the restoration of the endothelial glycocalyx and attenuation of inflammation as important properties for the choice of fluid. All studies were at potential risk of bias due to unclear randomization, concealment, and blinding. There were important threats to translatability for all studies. Heterogeneity precluded any meta-analysis. Another systematic review addressing the effects of volume resuscitation on the microcirculation in animal models of lipopolysaccharide sepsis was also unable to perform a meta-analysis because of the high heterogeneity of the studies [71].

Considering that the utilization of crystalloid balanced solutions might improve the outcome of critically ill patients [72], their effect on microcirculation is a relevant issue. In a sheep model of fecal peritonitis, the resuscitation with two balanced solutions, lactate Ringer solution or Plasmalyte® was associated with reductions in hyperchloremic acidosis, hemodynamic instability, organ dysfunctions and also with less sublingual microcirculatory derangements than with NaCl 0.9% [73]. Ringer acetate also improved intestinal, hepatic, and muscle microcirculation compared to saline solution in fecal peritonitis and hemorrhagic shock in mice [74, 75]. In contrast, another animal study did not show advantages of balanced solutions on microcirculation [76]. No clinical study has compared the effects of balanced crystalloid and saline solution on sublingual microcirculation.

## Is the sublingual mucosa an adequate window to assess the effects of fluids on microcirculation?

The sublingual mucosa is easily accessible for videomicroscopic evaluation. Videos can be acquired from this area with minimal discomfort for patients. Nevertheless, the relationship of sublingual microcirculation to other microvascular beds is controversial. In experimental models of hemorrhagic and endotoxemic shock, the reduction in cardiac output expectably results in compromise of every microvascular bed. Consequently, sublingual, intestinal, and renal microvascular flow can be correlated. For example, progressive bleeding reduced microvascular flow in sublingual mucosa and intestinal mucosa and serosa [77]. In porcine endotoxic shock, renal microvascular alterations measured by contrast-enhanced ultrasound imaging were mirrored by changes in sublingual microcirculation [78]. Similar findings were described in hemodilution [79] and during hemorrhagic shock and fluid resuscitation [80]. Notwithstanding these results, the intestinal mucosal and especially the peritubular microcirculation might be more severely compromised than sublingual mucosa [81, 82]. The lack of coherence among different territories may be more evident after the resuscitation [82, 83].

Similar dissociation was also found in clinical studies. The characteristics of sublingual microcirculation differed from that of a mucosal ostomy in patients with abdominal surgery [42, 84]. Fluid challenges recruited sublingual but not stoma mucosa in septic patients [42]. Moreover, patients’ outcomes were linked to intestinal but not sublingual microcirculation [42]. In septic patients, contrast-enhanced ultrasound showed decreased renal cortical perfusion that was not adequately reflected by changes in sublingual microcirculation [85]. Therefore, septic patients may exhibit intestinal and renal microvascular disorders with concomitant normal sublingual microvascular perfusion.

## Limitations of the studies

Some of the studies addressing the effects of fluids on sublingual microcirculation that were analyzed in this review have severe methodological weaknesses. In some cases, the analysis of microcirculatory videos has been performed by unreliable methodologies. These include an unspecified point of care evaluation [46], point of care assessment of microvascular flow index [68] and the use AVA 4.3 software for automatic analysis of microcirculation [26, 40, 45, 48]. The point of care analysis has a poor reproducibility compared to the quadrant or vessel-by vessel assessment [86, 87]. The AVA 4.3 software has never been validated. On the contrary, some studies consistently showed that the successive versions of AVA 4 software might be unreliable [88–90]. Compared to the reference method, the AVA 3.2 software-assisted analysis, AVA 4 shows wide 95% limits of agreement, and frequently results in values outside the physiological range for all microcirculatory variables [88, 89]. Moreover, AVA 4 failed to recognize microvascular changes induced by anesthesia and hemorrhagic shock [89, 90]. In addition, videos posted as typical examples in the supplementary material of some studies show severe compression artifacts [37, 39, 47]. These videos should have been deleted from the analysis. Severe methodological pitfalls such as compression artifacts and inadequate analysis preclude relevant conclusions about the effects of fluids on sublingual microcirculation. Research on microcirculation requires effort to overcome these drawbacks and generate sound scientific evidence.

## Conclusions

Clinical and experimental evidence suggest that fluids can improve sublingual microcirculation. This helpful effect seems dependent on the magnitude of cardiac output increase and on the occurrence of more severe tissue hypoperfusion. Despite theoretical considerations and scarce experimental evidence favoring colloid solutions, crystalloids seem clinically equivalent to recruit microcirculation. Neither there is robust evidence supporting advantages of balanced crystalloids over saline solution. In addition, the effects of fluids on sublingual microcirculation might differ from those on other microvascular beds, such as gut and kidneys. Considering the scarce evidence and the methodological limitations of the existing publications, high-quality studies are needed to definitively clarify the effects of fluids on microcirculation.

## Data Availability

Not applicable.

## Abbreviations

HES: Hydroxyethyl starch
PEG: Polyethylene glycol
